# Incidence of Postoperative Pain at 7 Days After Day Surgery Reported Using a Text Messaging Platform: Retrospective Observational Study

**DOI:** 10.2196/33276

**Published:** 2022-10-25

**Authors:** Vincent Compère, Alban Mauger, Etienne Allard, Thomas Clavier, Jean Selim, Emmanuel Besnier

**Affiliations:** 1 Department of Anesthesia and Intensive Care Rouen University Hospital Rouen France; 2 Inserm U982 Normandie University Mont-Saint-Aignan France; 3 Department of Anesthesia, Le Havre Hospital Montivilliers France

**Keywords:** day surgery, postoperative pain, emergency consultation, rehospitalization, ambulatory management, pain management, postsurgery, postoperative, ambulatory surgery, hospitalization, health care, mobile health, mobile platform

## Abstract

**Background:**

The most frequent complication observed after ambulatory surgery is acute postoperative pain.

**Objective:**

The purpose of this study was to evaluate the late incidence of postoperative pain at 7 days after day surgery.

**Methods:**

We retrospectively included patients who underwent day surgery under general or regional anesthesia and those who underwent local anesthesia in Rouen University Hospital from January 2018 to February 2020. Data collected were moderate-to-severe pain reports defined as numeric rating scale (NRS)>3/10 at 1 day (secondary end point) and 7 days (primary end point) after surgery. These data were collected using a semi-intelligent SMS text messaging platform to follow up with the patient at home after ambulatory surgery. Univariate and multivariate analyses were performed to analyze the risk factors for pain.

**Results:**

We analyzed 6099 patients. On the day after the surgery, 5.2% (318/6099) of the patients presented with moderate-to-severe pain: 5.9% (248/4187) in the general or regional anesthesia group and 3.7% (70/1912) in the local anesthesia group. At 7 days after the surgery, 18.6% (1135/6099) of the patients presented with moderate-to-severe pain, including 21.3% (892/4187) of the patients in the general or regional anesthesia group and 12.7% (243/1912) of the patients in the local anesthesia group. General surgery (odds ratio [OR] 1.54, 95% CI 1.23-1.92; *P*<.01) and orthopedic surgery (OR 1.66, 95% CI 1.42-1.94; *P*<.01) were associated with more late postoperative pain risk. Male gender (OR 0.66, 95% CI 0.57-0.76; *P*<.01), ophthalmology surgery (OR 0.51, 95% CI 0.42-0.62; *P*<.01), and gynecologic surgery (OR 0.67, 95% CI 0.50-0.88; *P*=.01) were associated with less late postoperative pain risk. The rate of emergency consultation or rehospitalization at 7 days after the surgery was 11.1% (679/6099). Late postoperative pain (OR 2.54, 95% CI 1.98-3.32; *P*<.001), general surgery (OR 2.15, 95% CI 1.65-2.81; *P*<.001), and urology surgery (OR 1.62, 95% CI 1.06-2.43; *P*=.02) increased the risk of emergency consultation or rehospitalization. Orthopedic surgery (OR 0.79, 95% CI 0.63-0.99; *P*=.04) and electroconvulsive therapy (OR 0.43, 95% CI 0.27-0.65; *P*<.001) were associated with less rates of emergency consultation or rehospitalization.

**Conclusions:**

Our study shows that postoperative pain at 7 days after ambulatory surgery was reported in more than 18% of the cases, which was also associated with an increase in the emergency consultation or rehospitalization rates.

## Introduction

Outpatient surgery represents a major challenge in the organization of care. The increasing number of outpatient surgeries highlights the need to ensure the highest level of safety for each patient. Establishing contact with patients after surgery is part of care in outpatient surgery, and it is strongly recommended by several practice guidelines to improve the management of postoperative complications at home [1]. This is increasingly done via automated information through SMS text message reminders. Several studies [2,3] have shown the benefit of using SMS text message reminders among patients with high blood pressure for decreasing their systolic blood pressure compared with usual care at 12 months and improvements in patients’ medication adherence. One study [4] developed a bank of text messages for the prevention of recurrent cardiovascular events. In another study [5], SMS text message reminders and a smartphone app were used successfully to monitor and reduce the alcohol consumption of military veterans—from a median of 5.6 units per drinking day in the first week to 4.7 units by the last week during the 4 weeks of study. For outpatient surgery, previous studies have shown that the use of SMS text message reminders before the surgery increased the rates of compliance with preoperative instructions [6], reduced the number of cancellations in gastrointestinal endoscopy [7], and decreased the rate of conversion to full-time hospitalization [8]. SMS text messaging might be an interesting alternative to follow patients at home during the postoperative period.

One of the adverse events observed after surgery is postoperative pain. A recent multicentric German cohort study of 50,523 inpatients found that pain scores on the first postoperative day were high, even though they were only minor surgical procedures frequently performed in outpatient surgery [9]. A few other studies [10-12] have shown that after day surgery, the incidence of moderate-to-severe postoperative pain ranged from 25% to 65%. These studies [10-12] sought to evaluate the pain at home after ambulatory surgery even though the analgesic strategies were better. There are French guidelines that highlight the importance of anticipating the management of postoperative pain [13]. These guidelines cover specific instructions from the beginning of anesthesia consultation with the prescription of a postoperative analgesic integrating a multimodal strategy, the precise times for taking the medication, and finally, the possible recourse in case of insufficient treatment [14]. In the intraoperative period, the emphasis is on strategies using infiltrations and peripheral blocks in addition to the multimodal analgesia strategy, including the use of nonsteroidal anti-inflammatory drugs [15,16]. In fact, a very recent study [17] on the latest technique of pain management in 2228 patients showed that only 7% of the patients rated their pain as more than 3/10 on the day after the surgery. However, studies have mainly focused on the early evaluation of pain (the first 3 or 4 days after the surgery) without always considering the occasional prolonged nature of this pain [18]. Time after surgery also influences the frequency and severity of pain following surgery. In a Dutch study conducted in 1490 surgical inpatients, 41% of the patients reported moderate or severe pain on the day of surgery, with a declining rate of 14% after 4 days [19]. Similarly, Peuchot et al [17] showed that the incidence of outpatients with pain at 7 days after the surgery was 2.60%. However, a recent study [8] showed that there are multiple distinct trajectories of acute postoperative pain intensity, with 63% of the hospitalized patients reporting high or moderate-to-highly stable and sustained pain in the first 7 days after the surgery. These postoperative pain trajectories were predominantly defined by patient factors and not surgical factors.

The purpose of this monocentric study was to evaluate the incidence of late postoperative pain at 7 days after day surgery via automated information with SMS text message reminders and to assess the risk factors for late pain occurrence.

## Methods

### Ethics Approval

Ethics approval for this study (ethics committee 2058568) was provided by the noninterventional research committee based at Rouen University Hospital in France (Chairperson Professor LM Joly, approval E2017-37), as per the French law. The requirement for written informed consent was waived by the committee.

### Study Design

We performed a retrospective study in the day surgery unit of the Rouen University Hospital from January 2018 to February 2020. All outpatients were included in this study. We included all patients who underwent regional or general anesthesia (we could not distinguish general anesthesia from regional anesthesia in the database) by an anesthesiologist and those who underwent local anesthesia by the surgeon for day surgery. Patients who did not show up for their surgery or did not receive the various SMS text messages sent at the time of the procedure were not included in this study.

### Enrolment Procedure

At the Rouen University Hospital, the patient’s day surgery pathway begins with a consultation with the anesthesiologist at least 48 hours before the surgical procedure for those receiving general or regional anesthesia. These patients receive an analgesic prescription at the end of the anesthesia consultation (to obtain analgesics at the pharmacy prior to the ambulatory surgery) and information concerning the use of analgesics during the same consultation [14]. The anesthesiologist insists on the importance of systematically taking the prescribed treatment postoperatively, even in the absence of pain. Two types of prescriptions are available depending on the expected postoperative pain. The anesthesiologist fills either Prescription A for moderate pain (expected numeric rating scale (NRS)>3, range 0-10) and Prescription B for severe pain (expected NRS> 6, range 0-10). Prescription A combines paracetamol-codeine (500 mg/30 mg) every 6 hours systematically during the first 2 days, which is extensible to 5 days (the quantity dispensed by the pharmacy was sufficient for 5 days) and ketoprofen (100 mg) every 12 hours systematically during the first 2 days maximum. Prescription B combines paracetamol-codeine (500 mg/30 mg) every 6 hours systematically during the first 2 days, which is extensible to 5 days (the quantity dispensed by the pharmacy was sufficient for 5 days), ketoprofen (100 mg) every 12 hours systematically during the first 2 days maximum, and morphine sulfate (10 mg) every 6 hours systematically for 2 days if NRS>6 (range 0-10). The local anesthesia group received the prescription of paracetamol-codeine (500 mg/30 mg) every 6 hours systematically during the first 2 days, which was extensible to 5 days (the quantity dispensed by the pharmacy was sufficient for 5 days). In both groups, information concerning the systematic administration of analgesics was indicated on the prescription.

General anesthesia was standardized in the operating theatre with the use of propofol for induction (2 mg/kg), total intravenous anesthesia of propofol (target between 2 µg/mL and 6 µg/mL) or sevoflurane (fraction of expired sevoflurane 2%) for the maintenance of hypnosis, and total intravenous anesthesia of remifentanil (target between 3 ng/mL and 6 ng/mL). Hyperalgesia was prevented using ketamine (0.15 mg/kg) during general anesthesia. Intraoperative analgesia was performed with routine administration of paracetamol (1 g), nefopam (20 mg), and ketoprofen (100 mg) in the absence of respective contraindications. Tramadol (50 mg) or morphine (0.1 mg/kg) administration was possible at the end of the procedure at the anesthesiologist’s discretion. During the patient’s hospitalization (postanesthesia care unit and outpatient surgery unit), pain was assessed using NRS. Morphine titration was performed in the postanesthesia care unit if necessary (NRS>3/10). Postoperative nausea and vomiting was prevented, as per the Apfel score, with perioperative dexamethasone (4 mg) and droperidol (1.25 mg). Intraoperative analgesia was also homogeneous with the administration of systematic paracetamol (1 g), nefopam (20 mg), and ketoprofen (100 mg) in the absence of contraindication and ropivacaine infiltration as soon as it was possible. In the local anesthesia group, local anesthesia protocols were standardized, including xylocaine (10 mg/mL) or ropivacaine (2 mg/mL) infiltration for all surgeries and oxybuprocaine eye drops for ophthalmology.

The process of the semi-intelligent platform of SMS text messaging has been previously described [17]. Messaging started 2 days before the surgery with an SMS text message reminder and the possibility of alerting the medical staff if the patient was unable to attend the surgery. The patient could respond ALERT if there was a medical problem or if assistance was required. A response different to ALERT was categorized as an unexpected response. There was no obligation to respond to the SMS text message reminder. On the day before the surgery, the patient received 3 messages informing about the required time of arrival and location of the outpatient unit, fasting recommendations, and hygiene rules. After surgery, patients received several SMS text message reminders with the possibility to answer. These were sent at 11 AM on the day after the ambulatory surgery in which the patient was asked successively if everything was fine, the intensity of pain determined by the NRS between 0 and 10, the presence of postoperative nausea and vomiting, or other medical-surgical complications. On the seventh day, the patient received another SMS text message asking about satisfaction with the management by using a numerical scale from 0 to 10, the need to have consulted the general practitioner, an emergency service, or to be rehospitalized in the context of the ambulatory surgery performed, as well as the pain felt and communicated using the numerical scale. The patients’ answers were centralized by Calmedica and then analyzed in an Excel spreadsheet software (version 2019, Microsoft).

### Primary and Secondary End Points

The primary end point was the incidence of maximum pain experienced by the patient whether at rest or on mobilization, defined by NRS>3 on a scale of 0 to 10 (0=no pain, 10=maximum pain) on the seventh day following ambulatory surgery. Secondary end points were the incidence of severe pain on day 7 (NRS>6), moderate (NRS>3) or severe pain (NRS>6) on day 1, the presence of postoperative nausea and vomiting on day 1, the incidence of emergency consultation or rehospitalization within 7 days after surgery as well as the risk factors for this variable, and overall satisfaction with the management on a scale of 0 to 10. Demographic data such as age and sex as well as the type of surgery were also collected.

### Statistical Analysis

Because of the retrospective and cohort nature of this study, we did not perform an a priori calculation of the sample size. The results are expressed as median and first and third quartile for quantitative data and as percentages for qualitative variables. The different data were compared with a chi-square test using the Prism software (version 6, GraphPad) for the qualitative variables. An overall analysis and then a subgroup analysis differentiating the “general or regional anesthesia” group and the “local anesthesia” group was performed. Univariate and multivariate analyses were performed with logistic regression models using the R software (version 3.0, R Core Team and the R Foundation for Statistical Computing). The variables included in the multivariate model were those clinically relevant, consistent with the literature, and with *P*<.90 in the univariate model. We performed simple imputation of the missing data by random draw with discounts for patients with consistent data. The only data transformation was the realization of age range to refine the fitting model. The evaluation of the model is represented by a figure representing the expected risk according to the observed risk of having the study criterion.

## Results

In this study, we analyzed the data of 6099 patients. The demographic, anesthesia regimen, and the surgical data of these patients are presented in Table 1.

**Figure 1:**
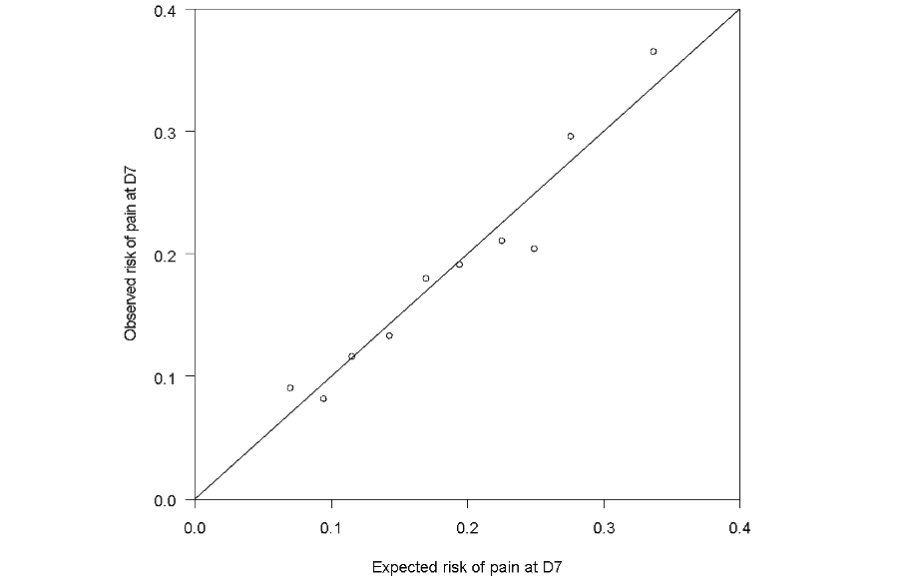
Multivariable calibration of pain risk (pain visual analog score >3) at 7 days after surgery. D7: 7 days after surgery.

On the day after the surgery, 5.2% (318/6099) of the patients presented with moderate-to-severe pain: 5.9% (248/4187) in the general or regional anesthesia group and 3.7% (70/1912) in the local anesthesia group. In the whole cohort, 1.4% (87/6099) of the patients expressed severe pain, whereas 1.5% (64/4187) of the patients in the general or local regional anesthesia group and 1.2% (23/1912) in the local anesthesia group expressed severe pain. Seven days after the surgery, 18.6% (1135/6099) of the patients presented with moderate-to-severe pain at 7 days after the day surgery: 21.3% (892/4187) of the patients in the general or regional anesthesia group and 12.7% (243/1912) in the local anesthesia group. In this cohort, 4.3% (265/6099) of the patients expressed severe pain (expressed by an NRS>6): 4.8% (201/4187) in the general or regional anesthesia group and 3.3% (64/1912) in the local anesthesia group. The results of the univariate analysis and the adjusted multivariate model are shown in Table 2 (the calibration of the multivariate model is shown in Figure 1).

**Figure 2:**
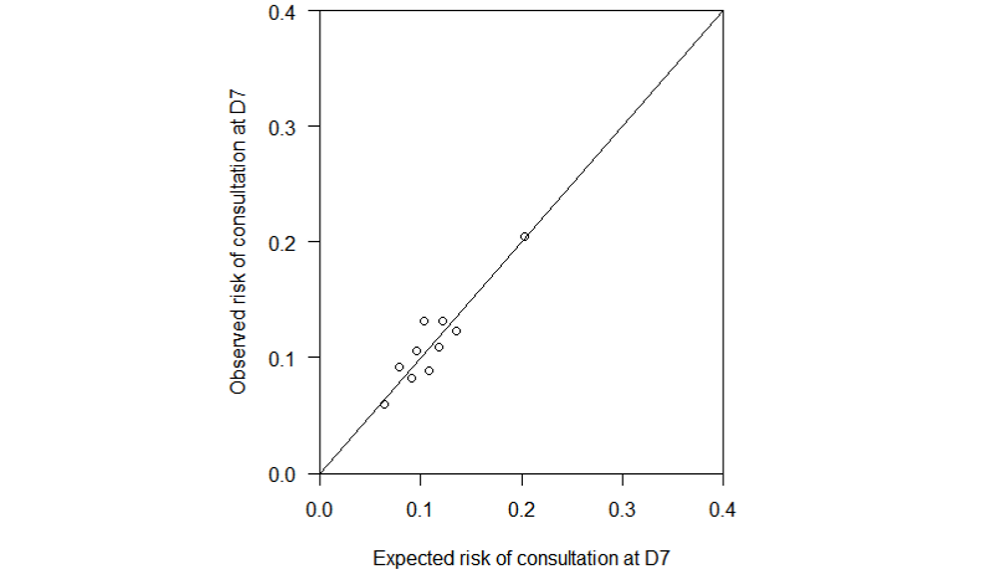
Multivariable calibration of emergency/rehospitalization risk at 7 days after surgery. D7: 7 days after surgery.

The incidence of emergency consultation/rehospitalization at 7 days after the surgery was 11.1% (679/6099). The results of the univariate analysis and the adjusted multivariate model for the risk factors are shown in Table 3 (the calibration of the multivariate model is shown in Figure 2).

The median global satisfaction was 9/10 [IQR 8-10] in the general or regional anesthesia group and 10/10 [IQR 8-10] in the local anesthesia group, with no significant difference between the 2 groups.

**Table 1:** Demographic, surgical, and anesthesia regimen data of the study population (N=6099).

Characteristics		Value
**Sex, n (%)**		
	Female	3004 (49.3)
	Male	2508 (41.1)
	Missing data	587 (9.6)
**Age (years)**		
	Mean (SD)	51 (19)
	Median (IQR)	54 (13)
**Age range (years), n (%)**		
	0-18	135 (2.2)
	18-40	1551 (25.4)
	40-60	1663 (27.3)
	60-70	1110 (18.2)
	70-80	736 (12.1)
	80-90	284 (4.7)
	>90	33 (0.5)
	Missing data	587 (9.6)
**Anesthesia procedure, n (%)**		
	General or regional anesthesia	4187 (68.7)
	Local anesthesia	1912 (31.3)
**Surgical discipline, n (%)**		
	Pneumology	372 (6.1)
	General surgery	424 (7)
	Ophthalmology	1620 (26.6)
	Orthopedic surgery	1172 (19.2)
	Plastic surgery	590 (9.7)
	Urology surgery	142 (2.3)
	Vascular surgery	339 (5.6)
	Gynecologic surgery	344 (5.6)
	Medical surgery	33 (0.5)
	Electroconvulsive therapy	365 (6)
	Otorhinolaryngology	105 (1.7)
	Missing data	593 (9.7)
**Postoperative nausea and vomiting** **, n (%)**		
	PONV^a^ D1^b^	57 (0.9)
	Pain NRS^c^>3 D1	318 (5.2)
	Pain NRS>6 D1	87 (1.4)
	Pain NRS>3 D7	1135 (18.6)
	Pain NRS>6 D7^d^	265 (4.3)
**Satisfaction scale at day 7 after the surgery, n (%)**		
	0-2	55 (0.9)
	3-5	169 (2.8)
	6-8	1221 (20)
	9-10	3711 (60.8)
	Missing data	943 (15.5)
Emergency consultation/hospitalization at 7 days after surgery, n (%)		679 (11.1)

^a^PONV: postoperative nausea and vomiting.

^b^D1: day 1 after surgery.

^c^NRS: numeric rating scale.

^d^D7: 7 days after surgery.

**Table 2:** Univariate and multivariate analysis of pain (pain numeric rating scale >3) at 7 days after surgery.

		Univariate		Multivariate adjusted	
		Odds ratio (95% CI)	*P* value	Odds ratio (95% CI)	*P* value
**Sex**					
	Female	1.00	N/A^a^	1.00	N/A
	Male	0.77 (0.61-0.8)	<.001	0.66 (0.57-0.76)	<.01
**Age (years)**					
	<18	1.00	N/A	1.00	N/A
	18-40	1.31 (0.86-2.09)	.23	1.36 (0.87-2.19)	.19
	40-60	1.39 (0.91-2.22)	.15	1.48 (0.94-2.41)	.10
	60-70	0.82 (0.53-1.33)	.40	1.05 (0.66-1.74)	.83
	70-80	0.75 (0.47-1.23)	.24	1.17 (0.72-1.97)	.53
	80-90	0.57 (0.33-1.01)	.05	1.02 (0.57-1.86)	.95
	>90	0.42 (0.10-1.30)	.18	0.63 (0.14-2.00)	.48
**Anesthesia procedure **					
	Local anesthesia	0.54 (0.46-0.63)	<.001	1.00	N/A
	General or regional anesthesia	1.00	N/A	1.18 (0.99-1.42)	.07
**Surgical discipline**					
	Pneumology	0.53 (0.38-0.75)	<.001	0.97 (0.74-1.25)	.80
	General surgery	1.00	N/A	1.54 (1.23-1.92)	<.01
	Ophthalmology	0.23 (0.17-0.30)	<.001	0.51 (0.42-0.62)	<.01
	Orthopedic surgery	1.02 (0.80-1.31)	.87	1.66 (1.42-1.94)	<.01
	Plastic surgery	0.57 (0.43-0.77)	<.001	0.99 (0.80-1.23)	.94
	Urology surgery	0.64 (0.40-0.99)	.05	0.86 (0.58-1.23)	.41
	Vascular surgery	0.47 (0.33-0.67)	<.001	0.93 (0.69-1.24)	.62
	Gynecologic surgery	0.54 (0.38-0.77)	.001	0.67 (0.50-0.88)	.01
	Medical surgery	0.79 (0.33-1.73)	.58	1.33 (0.60-2.67)	.44
	Electroconvulsive therapy	0.77 (0.56-1.07)	.12	1.23 (0.97-1.56)	.08
	Otorhinolaryngology	0.51 (0.29-0.87)	.02	0.92 (0.58-1.42)	.73

^a^N/A: not applicable

**Table 3:** Univariate and multivariate analysis of emergency/rehospitalization risk at 7 days after surgery.

			Univariate					Multivariate adjusted		
			Odds ratio (95% CI)		*P* value		Odds ratio (95% CI)			*P* value
**Sex**										
	Female	1		N/A^a^		1			N/A	
	Male	0.82 (0.70-0.96)		.02		0.87			.14	
**Age (years)**										
	<18	1		N/A		1			N/A	
	18-40	0.88 (0.54-1.51)		.63		1.15 (0.70-1.99)			.60	
	40-60	0.77 (0.48-1.32)		.32		1.22 (0.73-2.13)			.47	
	60-70	0.51 (0.30-0.88)		.01		0.82 (0.48-1.49)			.50	
	70-80	0.81 (0.48-1.41)		.43		1.35 (0.78-2.46)			.30	
	80-90	0.88 (0.54-1.51)		.63		1.56 (0.83-3.00)			.17	
	>90	0.84 (0.23-2.45)		.77		1.41 (0.70-1.99)			.57	
**Anesthesia procedure **										
	Local anesthesia	0.82 (0.69-0.98)		.03		0.93 (0.73-1.17)			.54	
	General or regional anesthesia									
**Surgical discipline**										
	Pneumology	0.87 (0.48-1.63)		.64		1.00 (0.54-1.96)			.99	
	General surgery	2.01 (1.57-2.55)		<.001		2.15 (1.65-2.81)			<.001	
	Ophthalmology	0.85 (0.70-1.03)		.09		0.82 (0.65-1.04)			<.10	
	Orthopedic surgery	0.77 (0.62-0.96)		.02		0.79 (0.63-0.99)			.04	
	Plastic surgery	0.87 (0.67-1.13)		.30		0.87 (0.65-1.15)			.33	
	Urology surgery	1.54 (1.02-2.27)		.03		1.62 (1.06-2.43)			.02	
	Vascular surgery	0.84 (0.59-1.16)		.30		0.87 (0.59-1.25)			.45	
	Gynecologic surgery	1.04 (0.76-1.40)		.79		0.99 (0.69-1.40)			.96	
	Medical surgery	1.03 (0.34-2.42)		.94		1.04 (0.34-2.45)			.93	
	Electroconvulsive therapy	0.49 (0.32-0.71)		<.001		0.43 (0.27-0.65)			<.001	
	Otorhinolaryngology	1.22 (0.72-1.96)		.64		1.00 (0.54-1.96)			.99	
PONV^b^ at day 1 after surgery			1.50 (0.69-2.93)		.26		N/A			N/A
Pain NRS^c^ >3 at day 7 after surgery			3.66 (2.98-4.39)		<.001		2.54 (1.98-3.32)			<.001

^a^N/A: not applicable.

^b^PONV: postoperative nausea and vomiting.

^c^NRS: numeric rating scale.

## Discussion

### Principal Findings

In this study on 6099 patients, on the day after surgery, 5.2% (318/6099) of the patients presented with moderate-to-severe pain: 5.9% (248/4187) in the general or regional anesthesia group and 3.7% (70/1912) in the local anesthesia group. At 7 days after the surgery, 18.6% (1135/6099) of the patients presented with moderate-to-severe pain, including 21.3% (892/4187) of the patients in the general or regional anesthesia group and 12.7% (243/1912) in the local anesthesia group. General surgery (odds ratio [OR] 1.54, 95% CI 1.23-1.92) or orthopedic surgery (OR 1.66, 95% CI 1.42-1.94) were associated with more late postoperative pain risk. The rate of emergency consultation or rehospitalization at 7 days after the surgery was 11.1% (679/6099) in this study. Late postoperative pain increased the risk of emergency consultation or rehospitalization (OR 2.54, 95% CI 1.98-3.32).

### Comparison With Prior Work

In this retrospective study, the prevalence of pain on the day after day surgery (318/6099, 5.2%) was similar to that found in a previous study (7%) [17] but much lower than that reported in the majority of the older studies. In a review published in 2002, the incidence of acute postoperative pain was 45% and ranged from 6% to 95% in 13 studies that included mixed surgical procedures [8]. McGrath et al [12] in 2004 found an acute postoperative pain incidence rate of 30% in a cohort of 5703 patients. In that work, the proportion of types of surgery was different from ours, with almost half of them being ophthalmologic surgery, which does not cause much postoperative pain. In a 2007 study, the mean visual analog scale scores were greater than 40 mm in 21% (119/648) of the patients at postoperative day 1 [11]. Our study included more than 50% of general and orthopedic surgery cases. In a very recent work that included 1691 patients, 35.5% of the patients reported moderate-to-severe pain at postoperative day 1 [20]. The low rate observed in our work could be explained by the high proportion of patients receiving local anesthesia for minor surgery (1912/6099, 31.3%). Indeed, in our study, 5.9% (248/4187) of the patients in the general or regional anesthesia group and 3.7% (70/1912) of the patients in the local anesthesia group presented with moderate-to-severe pain. The decrease in the proportion of patients with acute postoperative pain is probably related to the improvement in pain management. The new advancements in the analgesia strategy used in our day surgery unit combine 3 main key components when not contraindicated: regional/local analgesia, acetaminophen, and nonsteroidal anti-inflammatory drugs [21]. This approach combined with education about postoperative pain integrated in enhanced recovery programs has been shown to improve surgical outcomes [22]. In our previous study of a randomized controlled trial in 186 patients, preoperative analgesic instruction and prescription during anesthesia consultation was found to reduce the incidence of early postoperative home pain in outpatient surgery from 48% to 24% on postoperative day 1 for surgery that generally results in severe postoperative pain [14].

Surprisingly, the prevalence of pain in this study increased from 5.2% (318/6099) to 18.6% (1135/6099) at 7 days after surgery in the general or regional anesthesia group (892/4187, 21.3%) more frequently than after that in the local anesthesia group (243/1912, 12.7%). This result is in agreement with 29.1% of the patients reporting moderate-to-severe pain at postoperative day 7 in the study of Carlier et al [20]. In another study on 1490 surgical inpatients, 41% of the patients reported moderate or severe pain on the day of surgery, with a declining rate of 14% after 4 days [19]. In the study of Gramke et al [11] on outpatients, the mean visual analog scale scores were greater than 40 mm in 21% of the patients on postoperative day 1, 13% on postoperative day 2, 10% on postoperative day 3, and 9% of the patients on postoperative day 4. In a previous work, we observed the passage from a rate of 7% at postoperative day 1 to 2.60% at postoperative day 7 [17]. The main difference between that work and this study is the surgical specialties included. Our previous work included more gynecological surgeries, which seemed to result in less postoperative pain in our multivariate analysis (OR 0.67, 95% CI 0.50-0.88), whereas in accordance with literature [9,12], general (OR 1.54, 95% CI 1.23-1.92) or orthopedic surgery (OR 1.66, 95% CI 1.42-1.94) is associated with more late postoperative pain risk. These 2 surgery disciplines represent more than 25% of the patients in our study. Barry et al [23] found that rebound pain (defined as an increase from well-controlled to severe pain typically within 12-24 hours of resolution of the nerve block) occurred in 49.6% of the cohort of 972 patients. In our study, 19.2% (1172/6099) of the orthopedic surgery cases could explain in part this higher prevalence of the late postoperative pain observed. Male gender was found to be a protective factor in our work (OR 0.66, 95% CI 0.57-0.76). This result is comparable with that of other studies that have identified female gender as a postoperative pain risk factor in both inpatient and outpatient settings. A very recent study [8] showed that the pain trajectory was more dependent on patient-related parameters than on those associated with the surgery, with young age and female sex being found as risk factors, as well as higher anxiety (OR 1.08, 95% CI, 1.01-1.14) and more pain behaviors (OR 1.10, 95% CI 1.02-1.18).

The emergency consultation or rehospitalization rate found in this study (679/6099, 11.1%) is higher than that reported in our previous work (6.7%) [17] but is comparable to that found by McIsaac et al in 2015 [24], wherein in their population-based cohort of 296,497 outpatients in Canada, 10.5% returned to the emergency unit or were readmitted to hospital within 30 days after the surgery. In another study [18], 2% of 744 patients were admitted to the hospital on an unplanned basis, returned to the hospital, or visited their general practitioner during the postoperative course, but the study was performed on 4 postoperative days only. Further, in a Danish multicenter study of morbidity after 57,709 day surgery procedures, the overall rate of return hospital visits was only 1.21% [25]. In the United Kingdom and Ireland pediatric units, the percentages of unplanned admissions varied from 0% to 16.3% in 93 participating centers [26]. The type of surgery could explain those results. In our study, general (OR 2.15 95% CI 1.65-2.81) and urology surgery (OR 1.62 95% CI 1.06-2.43) were identified as risk factors, whereas orthopedic surgery seemed to be a protective factor (OR 0.79, 95% CI 0.63-0.99). In our unit, patients were systematically reviewed in consultation by an orthopedic surgeon in the week following surgery, a process which probably makes the postoperative course safer. Finally, we observed that postoperative pain increases the risk of emergency consultation or rehospitalization (OR 2.54, 95% CI 1.98-3.32). McGrath et al [12] showed that postoperative pain is one of the reasons for nurse or physician consultation, unplanned consultation, or hospital readmission. Pain was the most commonly reported reason for return, occurring in 38% of the patients who had an unanticipated admission or in 20,817 patients requiring readmission. The general surgery service had the highest rate of unanticipated admissions or readmissions (3.2%), followed by otorhinolaryngology (3.1%) and urology (2.9%) clinics [27].

### Strengths and Limitations

There are several limitations in this study. First, this study had a retrospective single-center design. However, the standardized organization of care teams was ensured because it was conducted in an outpatient surgery unit of a university hospital center. Second, we have no data on the different parameters. Patients were not identified as per the anesthesia regimen between general or regional anesthesia. Ophthalmology is also an important contributor to the number of local anesthesia cohorts, thereby leading to possible bias. The postdischarge medical adherence was not assessed in our study and may have contributed to the postoperative pain scores. Third, we did not measure patient perceptions with an adapted skill instrument. Multidimensional scales have now been developed and appear to be more relevant than a simple numerical scale [28,29]. Recent developments in the assessment of quality parameters after surgery have led to the implementation of quality of recovery as a principal end point after day case surgery. The quality of recovery is related to various aspects of patients’ daily living after discharge to home.

In conclusion, this work shows that postoperative pain at 7 days after ambulatory surgery, integrated with new advancements in the management of postoperative analgesia (using infiltrations and peripheral blocks in addition to multimodal analgesia, including the use of nonsteroidal anti-inflammatory drugs), was reported in more than 18% of the cases, which is also associated with an increase in the emergency consultation or rehospitalization rates.

## Abbreviations

NRS: numeric rating scale
OR: odds ratio
